# Opportunity or catastrophe? effect of sea salt on host-parasite survival and reproduction

**DOI:** 10.1371/journal.pntd.0009524

**Published:** 2022-02-24

**Authors:** Ao Yu, J. Trevor. Vannatta, Stephanie O. Gutierrez, Dennis J. Minchella

**Affiliations:** Purdue University, West Lafayette, Indiana, United States of America; University of Oxford, UNITED KINGDOM

## Abstract

Seawater intrusion associated with decreasing groundwater levels and rising seawater levels may affect freshwater species and their parasites. While brackish water certainly impacts freshwater systems globally, its impact on disease transmission is largely unknown. This study examined the effect of artificial seawater on host-parasite interactions using a freshwater snail host, *Biomphalaria alexandrina*, and the human trematode parasite *Schistosoma mansoni*. To evaluate the impact of increasing salinity on disease transmission four variables were analyzed: snail survival, snail reproduction, infection prevalence, and the survival of the parasite infective stage (cercariae). We found a decrease in snail survival, snail egg mass production, and snail infection prevalence as salinity increases. However, cercarial survival peaked at an intermediate salinity value. Our results suggest that seawater intrusion into freshwaters has the potential to decrease schistosome transmission to humans.

## Author summary

Climate change can impact host-parasite systems by rising seawater levels, which flood coastal regions and increase salinity in many freshwaters. Host-parasite interactions are a key component of freshwater ecosystems, however, the effects of sea water intrusion on host-parasite dynamics are largely unknown. In this study, we quantify the effects of sea salt concentration on the model host-parasite system, *Biomphalaria alexandrina* and *Schistosoma mansoni*. We demonstrate a significant, negative relationship between sea salt concentration and host survival, host reproduction, and parasite success. The increase in freshwater salinity associated with sea level rise has the potential to decrease parasite transmission and disease burden in humans and wildlife.

## Introduction

Anthropogenic climate change, associated with increasing greenhouse gases, can change many environmental factors including temperature, pH, precipitation, as well as salinity in both terrestrial and aquatic systems. Increasing salinity in freshwater systems is often caused by rising sea levels and seawater intrusion [1,2]. Salt concentration (salinity) is a crucial abiotic factor that impacts many aspects of biotic interactions [3]. Increases in salinity of freshwaters will undoubtedly result in changes in organismal growth, reproduction, and survival, impacting entire food webs, and thus host-parasite interactions.

Parasites play a key role in freshwater communities [4], and they are commonly recognized for their ability to modify the growth, reproduction, and survival of their hosts [5]. Parasites are also the etiologic agents of human disease. Therefore, understanding how abiotic factors and parasitic diseases interact in the context of climate change, more specifically with increasing salinity, is critical in order to understand human disease transmission.

Rising seawater levels and seawater intrusion affect numerous bodies of water including the Nile River Delta. The salinization of coastal land in the Nile Delta is caused by the decrease in the Nile River’s freshwater levels due to human activity and the increasing sea levels in the Mediterranean Sea [6]. Seawater will submerge large areas in the coastal zone of the Nile Delta in the near future, impacting host-parasite interactions in the region [7].One parasitic disease of humans acquired in the Nile Delta of Egypt is schistosomiasis, which is caused by the trematode *Schistosoma mansoni* [8].

*Schistosoma mansoni* completes its life cycle by alternating between two hosts: intermediate host snails and definitive hosts such as humans. Parasite eggs hatch in water, becoming the first stage parasite, called miracidia, which infect snails. The parasites develop in the infected snail hosts into secondary parasite larvae, called cercariae, which are then released into freshwaters. Parasite transmission occurs when humans come in contact with cercariae which burrow into human skin. Adult worms develop and reproduce in the human host. The life cycle is completed when parasite eggs in human feces are released back into freshwater. There is no vaccine currently available for *Schistosoma* infection. Current control strategies for this disease involve curing patients by administration of praziquantel [9] and biological or chemical controls aimed at reducing intermediate host snail populations [10,11].

Approximately, 12.7 million infected people are clustered in the Middle East and North Africa region, and Egypt’s share of the infected population is about 7.2 million [12]. In areas with perennial irrigation such as the Nile Delta and the Nile River Valley, schistosomiasis prevalence is high (60% infection rate) [13]. In contrast, the infection rate is relatively low (6%) in districts of basin irrigation (commonly known as annual flooding) [13]. Development and shift from basin irrigation to perennial irrigation in Egypt has resulted in year-round availability of water in many districts making the likelihood of infection high [14], but the impact of seawater intrusion on parasite transmission in this system is unclear.

This laboratory study explores the effect of increasing salinity (as artificial seawater) on host and parasite success using the Egyptian strain of *Schistosoma mansoni* and its snail intermediate host, *Biomphalaria alexandrina*, simulating the conditions in the Nile Delta of Egypt. *B*. *alexandrina* is ubiquitous in freshwater habitats of the Nile Delta [15]. In order to assess the success of parasite transmission, we exposed snail hosts to the trematode parasite and monitored snail survival, snail egg mass production, infection prevalence, and parasite (cercarial) survival. We predicted lower snail survival and egg mass production as snails become stressed by osmoregulation and allocate less energy to reproduction [16,17]. We further expected to observe lower parasite infection prevalence in snails as salt concentration increased, since the parasite larvae that infect snails (miracidia) may be less successful due to increased energy expenditures required to maintain osmolarity [18,19]. Finally, we predicted lower cercarial (the parasite larvae which infects humans) survival as salt concentration increased due to increases in energy expenditures on osmoregulation [20] and fewer available resources from stressed hosts [21].

## Materials and methods

### Ethics statement

Husbandry and euthanasia of mice was reviewed and approved by the Purdue Animal Care and Use Committee (PACUC Protocol # 1111000225) in order to isolate *Schistosoma* eggs.

### Experimental reagents

Salinity of the Mediterranean Sea is approximately 38 parts per thousand (ppt) [22], which we considered saturated seawater (100% salt solution) in this experiment. The Nile Delta is composed of multiple water bodies so it would be challenging to determine a single value for the salinity of the Nile Delta. However, Burullus Lake and Manzala Lake, two well studied lakes in the Nile Delta region, have a high prevalence of *S*. *mansoni* infection [23]. Burullus Lake has salinity levels ranging from 2.1 ppt to 17.2 ppt from the west to the north [24], with an average around 3.0 ppt throughout the year [25]. Manzala lake has a salinity value that varies between 0 to up to 35 ppt [26]. Previous studies have shown that *B*. *alexandrina* can avoid negative fitness consequences of high salinities to a certain extent by retreating to areas of freshwater input [27] but, in general, are unable to survive salinities above 7.6 ppt [28,29]. Salinity treatment groups in our study consisted of 1% (0.38 ppt), 5% (1.9 ppt), 10% (3.8 ppt), and 15% (5.7 ppt) seawater solutions, which were chosen based on additional pilot data suggesting seawater concentrations of 20% (7.6 ppt) and higher rapidly killed snail hosts. Our pilot study was intended to find the maximum salinity at which snails could survive. Therefore, the chosen salinity treatments represent the range of salinity expected in the Nile Delta in which snail survival and reproduction would still be likely. As part of our pilot study, various sea salt brands were assessed to determine which most closely resembled the mineral concentrations of seawater (supplement **Fig A** and **Table A in S1 Text**). Based on these comparisons, Instant Ocean Sea Salt (Spectrum Brands) was used to replicate seawater.

The four salinity treatments were prepared 2 to 3 days before usage from 100% stock solution (38 ppt) diluted with well water (the 1% solution), and the salinities of the final solutions were verified by using a handheld refractometer (Premium Aquatics). The well water used in this experiment comes from an unchlorinated well used by Purdue University for research and is periodically monitored by the university for contaminants. The final solutions were not aerated artificially but only aerated by natural gas exchange with the air. The 1% (0.38 ppt) salinity treatment was designated as the control for this experiment as this was the salinity of well water used for dilutions and matches the lowest salinities found in the Nile Delta.

### Experimental strain maintenance

We used *Biomphalaria alexandrina* snails and *Schistosoma mansoni* parasites, which both originated from Egypt. *B*. *alexandrina* snails were born and raised under controlled laboratory conditions (~25°C the ambient temperature of the laboratory, 12-hour light/12-hour dark, water salinity = 0.38 ppt). The *S*. *mansoni* life cycle was maintained for the experiment using *B*. *alexandrina* snails and male Balb/c mice [30].

In order to avoid spurious results associated with sudden seawater exposure, 60 snails were placed directly into each of the salinity treatments and acclimated for five days (total of 240 snails). This resulted in 60 snails each in the 0.38 ppt and 1.9 ppt, 46 snails in the 3.8 ppt, and 59 snails in the 5.7 ppt treatment. Surviving snails (total of 225 snails) were considered acclimated to the treatment conditions and included in this study for subsequent *Schistosoma* infections. During the experiment, snails were housed in individual 225 mL jars with a 2cm-by-2cm piece of Styrofoam (for egg laying) and were fed romaine lettuce *ad libitum*. Each jar was covered with glass plates that allowed air exchange but limited evaporation. Jars were not aerated, but water in each jar was changed weekly to maintain salinity levels. Weekly water changes are sufficient for snail maintenance given their small volume [31,32]. Pilot data showed no change in salinity due to evaporation over this period. During weekly water changes, egg masses were counted and removed from jars. While individual eggs were not counted, previous studies have demonstrated a strong correlation between number of eggs and number of egg masses [33,34].

### Snail infections

The experiment was conducted during the 12-hour light period. Mice were euthanized in accordance with Purdue Animal Care and Use Committee Protocol # 1111000225 to isolate *Schistosoma* eggs. The collected eggs were placed in freshwater (0.38 ppt) for approximately 60 minutes to allow the miracidia (the infective stage for the snails) to hatch. The resulting miracidia were transferred to well plates containing snails and 10 mL of their respective salinity treatment. Each snail was exposed individually using eight miracidia for 18 hours in the corresponding salinity treatment. Independent observations of miracidia were made, and miracidia were seen swimming normally after 1 hour in the highest salinity treatment. Due to the short lifespan of miracidia, we were unable to acclimate these parasites to salinity treatments before experimental infections. As such, our experiment is unable to distinguish between parasite infectivity and host susceptibility. Thus, we refer to the combined effect of host susceptibility and parasite infectivity simply as salinity impacts on infection prevalence.

### Data collection

Snail survival and egg masses laid were recorded weekly for eight weeks. Snail mortality was assessed by whether the snail was motile, responded to a stimulus, or was obviously decaying (empty shell). In cases where mortality was uncertain, the snail’s head-foot was touched with a blunt probe. If no response was seen, this snail was defined as dead. Snail egg masses were counted by checking the Styrofoam and the sides of the jar. Styrofoam on the water surface of each jar was removed with a forceps. Snail egg masses on the Styrofoam were counted and scraped off before placing the Styrofoam back. Sides of the jar were checked for snail egg masses, and these were scraped off before water changes with prepared salinity treatments.

In weeks four to seven post-parasite exposure, snails from each group were transferred into well plates filled with 10 mL of corresponding salinity treatment solution and cercarial release was observed. After one hour under artificial light, we examined the entire well first for the presence of cercariae. Then, 1 mL of mixed solution was drawn from each well containing cercariae, and the number of cercariae was counted for each snail [31]. Snails were checked for cercariae often (285, 280, 187, and 196 times for the 0.38 ppt, 1.9 ppt, 3.8 ppt, 5.7 ppt salinity treatments respectively, resulting in a total of 948 observations). Snails in which at least 10 cercariae were detected from the randomized 1 mL out of 10 mL were designated as infected while snails that produced less than 10 cercariae were designated as uninfected. Out of 948 observations only 13(1.37%) represented snails that were assigned as uninfected despite having countable cercariae from the randomized 1 mL sample. Thus, cross-contamination or extremely low intensity infections were infrequent, and likely had little impact on our results.

Eight weeks after parasite exposure, cercariae from the 0.38 ppt salinity (control) treatment were collected and used to examine cercarial survival in various salt solutions. Cercarial survival was assessed using only cercariae from the 0.38 ppt salinity treatment due to high snail mortality and low infection prevalence in the other treatment groups. Cercaria mortality was assessed by whether the cercaria was motile, responded to a stimulus, or was obviously disformed (disconnected head and tail). In cases where mortality was uncertain, the water around the cercariae was disturbed with a micropipette tip. If no response was seen, this larva was defined as dead. A large portion of the dead cercariae in this experiment had the head and tail disconnected, which aided in this determination. A total of 2709 cercariae were utilized in the cercarial survival study. Approximately 20 cercariae were placed into individual wells with 1ml of the four salinity treatment solutions. Cercariae survival was checked at 4h, 8h, 12h, and 24h after release from the snail, and cercariae were removed if little to no movement was detected [35,36]. Following the 24h check, all cercariae were euthanized with ethanol and counted to determine the exact number of cercariae in each trial. Cercariae have a period of maximum infectivity around 3–5 hours post-emergence. Parasite infectivity decreases by 50% at 8 h [37] and by 90% around 14–15 hours post-emergence [37]. Therefore, the time points of 4, 8, 12, and 24 hours would have the greatest biological relevance. Some cercariae can survive for more than 24 hours, but viability decreases considerably after this point [38–40]. The graphical abstract provides an overview of the experimental design and data collection.

### Statistical analyses

Snail survival was analyzed with a proportional hazard regression model using the survminer and survival packages in R [41,42]. Snail egg mass production was only compared between uninfected snails to remove the confounding influence of parasitic castration. Even after removal of infected snails, the data had considerable zero-inflation. To account for this, we used a zero-inflated, negative binomial, mixed effects model created in the glmmTMB package to analyze snail reproduction [43]. Differences in infection prevalence between salinity treatments were examined using a binomial General Linear Model. Cercarial survival was analyzed with the coxme package using a mixed effects proportional hazard model [44]. This was done to control for non-independence as multiple cercariae were taken from the same snail. In these models, salinity treatment was used as a fixed factor and individual snail ID was used as a random factor. All pairwise comparisons were made using the emmeans package with the multivariate t distribution for p value correction [45]. All analyses were performed in R version 3.6.3 [46].

## Results

### Effect of seawater on snail survival

**Fig 1:**
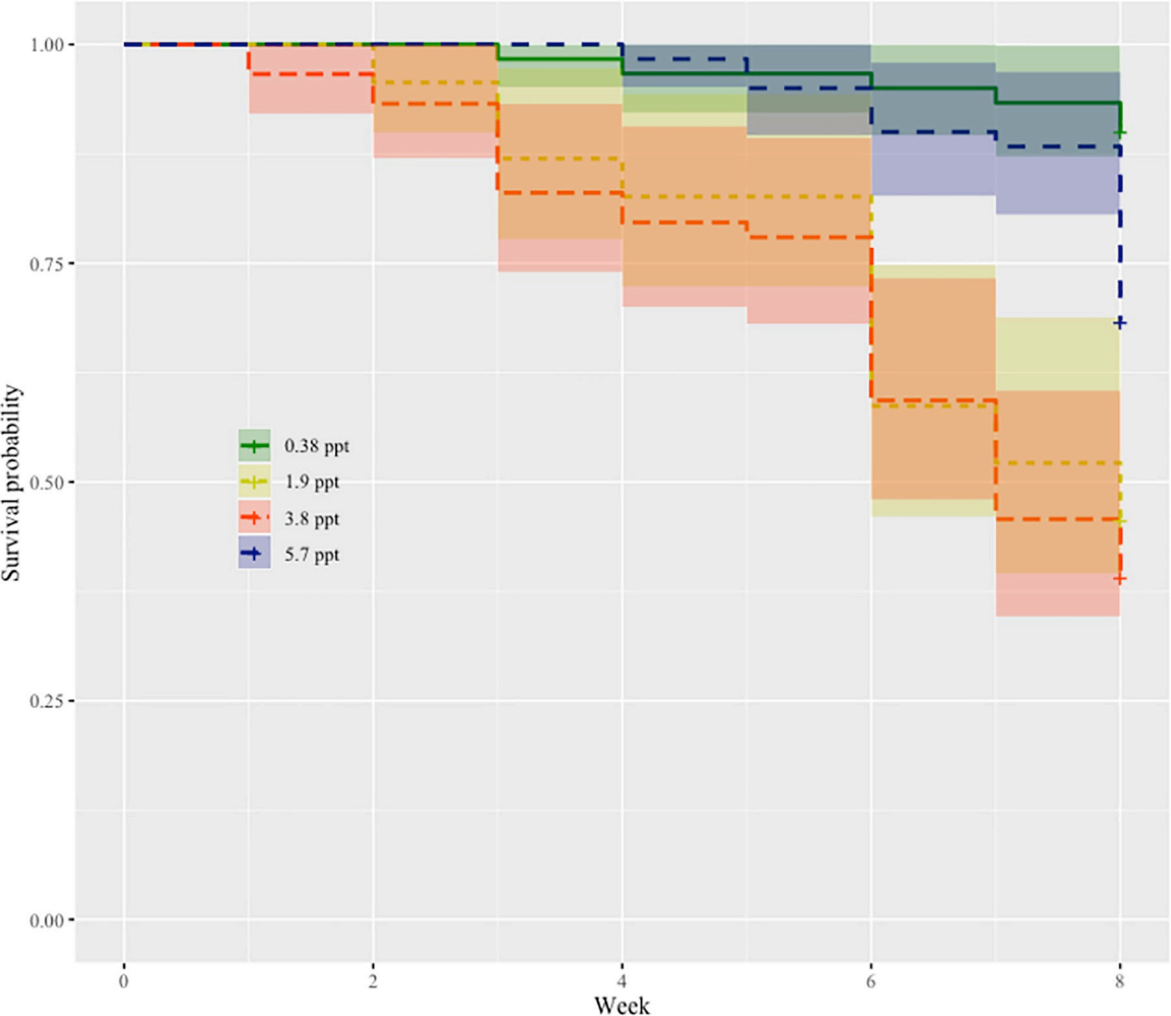
Probability of survival for snails in treatment groups of 0.38 ppt, 1.9 ppt, 3.8 ppt, 5.7 ppt salinity treatments. Snail survival in 0.38 ppt is significantly higher than 1.9 ppt (p < 0.05), 3.8 ppt (p < 0.0001), and 5.7 ppt (p < 0.0001). The survival probability of 1.9 ppt is significantly higher than 3.8 ppt and 5.7 ppt salinity treatments (p < 0.05). For coefficients and pairwise comparisons see supplement **Table B in S1 Text**.

Survival probability of infected and uninfected snails in the 0.38 ppt control treatment did not differ significantly over the 8-week experiment (p > 0.05; **Fig B in S1 Text**). Therefore, we compared snail survival probability across all salinity treatments regardless of infection status (**Fig 1**). All salinity treatments were significantly different from each other except for 3.8 ppt and 5.7 ppt (p > 0.05). Snails in the 0.38 ppt control treatment had the highest survival probability with a hazard ratio greater than seven times lower than 3.8 (p < 0.0001) or 5.7 ppt (p <0.0001), while the 3.8 ppt and 5.7 ppt salinity treatments had lower survival than the 1.9 ppt salinity treatment (p < 0.05), with hazard ratios 2 to 3 times higher than the 1.9 ppt treatment (**Fig 1;** For coefficients and pairwise comparisons see supplement **Table B in S1 Text**).

### Effect of seawater on snail egg production

**Fig 2:**
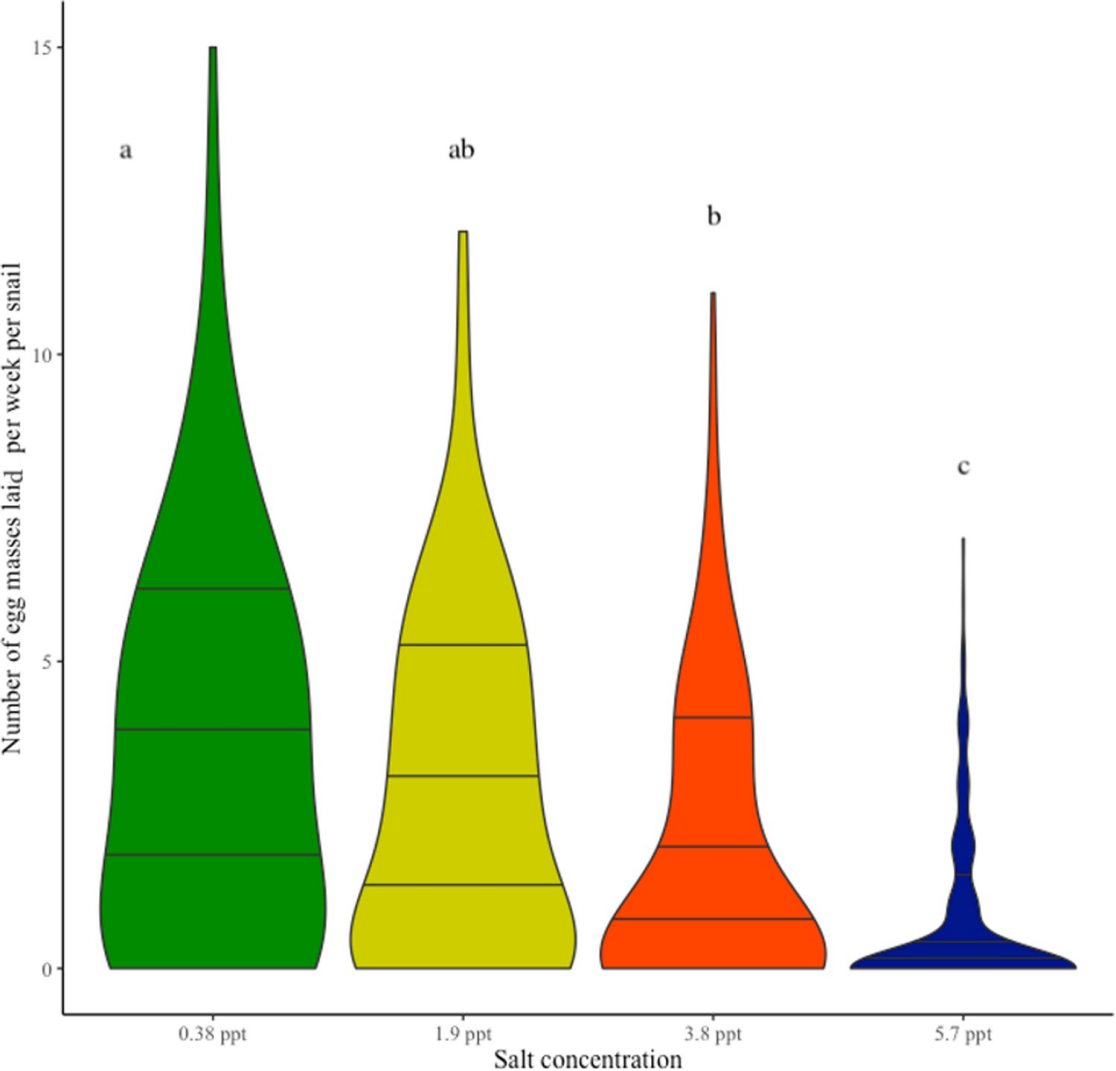
Egg mass output for uninfected snails in treatment groups 0.38 ppt, 1.9 ppt, 3.8 ppt, and 5.7 ppt. The sample size for uninfected snails are 18 snails in 0.38 ppt with 144 total observations, 47 snails in 1.9 ppt with 376 total observations, 33 snails in 3.8 ppt with 264 total observations, and 39 snails in 5.7 ppt with 312 total observations. Snails in the 0.38 ppt salinity treatment had significantly higher egg mass output than 3.8 ppt (p < 0.05) or 5.7 ppt (p < 0.0001). Snails in the 1.9 ppt and 3.8 ppt treatments also had a higher output compared to the 5.7 ppt treatment (p < 0.0001 and p < 0.05, respectively). The horizontal lines on the violin plot represent data quantiles of 25%, 50%, and 75%. Letters above bars indicate significant differences in egg mass production, with different letters representing significant differences. For pairwise comparisons see supplement **Table C in S1 Text**.

Snails infected with *Schistosoma mansoni* eventually become castrated and cease producing egg masses [47]. In our experiment, all but two infected snails in the 0.38 ppt salinity treatment were completely castrated by week 8 of the experiment. Since parasite infection will gradually lower egg mass production, only uninfected snails, ones that failed to produce cercariae after miracidia exposure, are analyzed here (supplemental analysis of infected snails suggests no appreciable differences in reproductive output across salinity treatments, **Fig C** and **Table D in S1 Text**). Thus, the decrease in egg mass production was likely in response to increases in salinity (**Fig 2**). Snails from the 0.38 ppt control treatment had higher egg output than snails in the 3.8 ppt (p < 0.05) and 5.7 ppt (p < 0.0001) salinity treatments. The 1.9 ppt treatment had a higher output than 5.7 ppt (p < 0.0001), and 3.8 ppt has a higher output than 5.7 ppt (p < 0.05). For coefficients and pairwise comparisons see supplement **Table C in S1 Text**.

### Effect of seawater on snail infection prevalence

**Fig 3:**
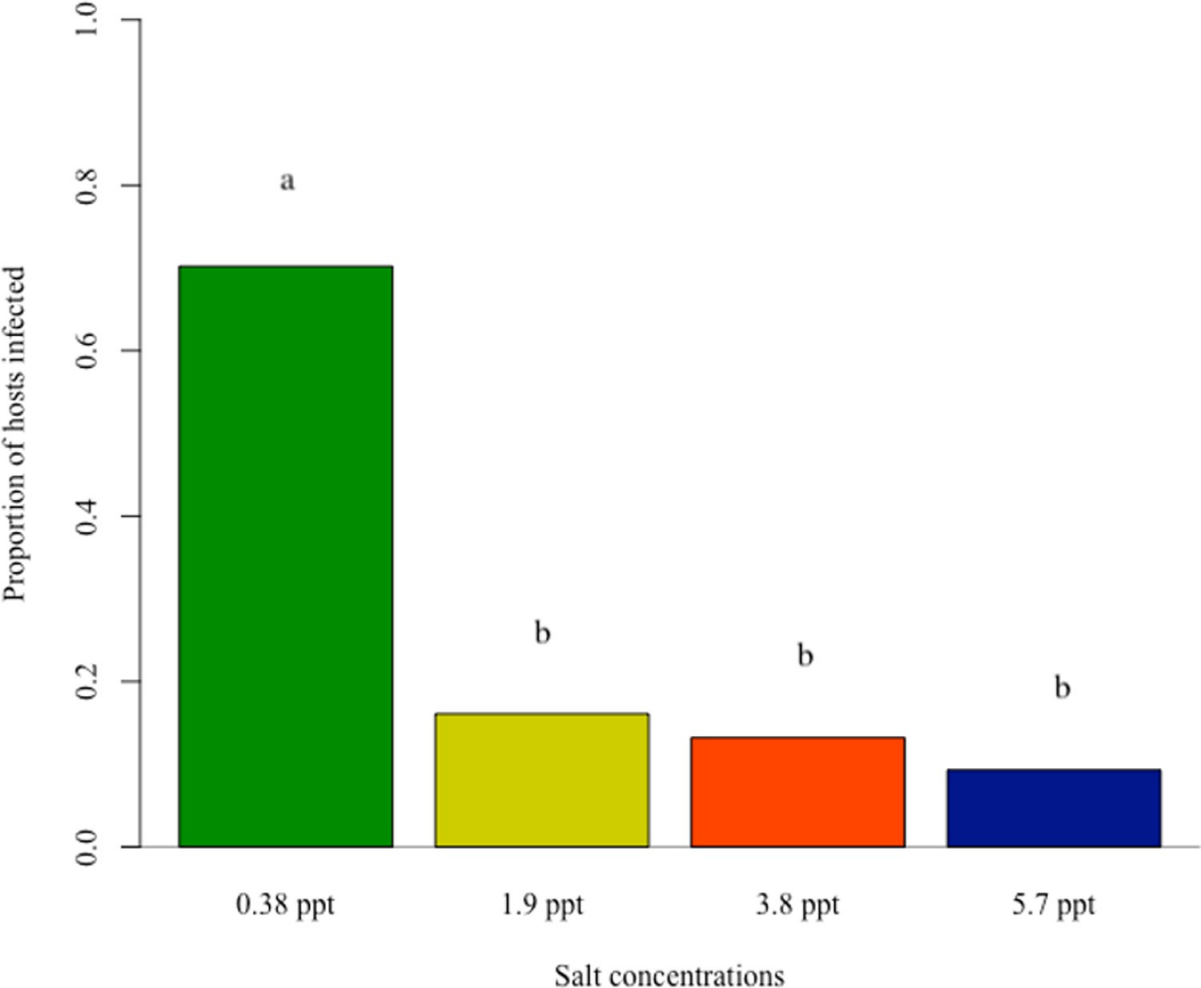
Snail infection prevalence in 0.38 ppt, 1.9 ppt, 3.8 ppt, and 5.7 ppt salinity treatments after exposure to *Schistosoma mansoni* miracidia. Different letters above bars indicate significant differences in infection prevalence among the treatments. For pairwise comparisons see supplement **Table E in S1 Text**.

Infection prevalence differed among the salinity treatments with a 70% prevalence in the 0.38 ppt treatment, 16% for 1.9 ppt treatment, 13% for 3.8 ppt treatment, and 9% for 5.7 ppt treatment. Snail infection prevalence in the 0.38 ppt control treatment had significantly higher prevalence than the other 3 treatments (p < 0.0001; For coefficients and pairwise comparisons see supplement **Table E in S1 Text**). However, the prevalence levels in the 1.9 ppt, 3.8 ppt, and 5.7 ppt salinity treatments were not significantly different from each other (p > 0.05) (**Fig 3**). Snail cercariae output counts were also recorded and suggest that increasing salinity leads to a reduction in cercarial output, but due to lower infection prevalence in the higher salinity treatment groups, this data should be treated as preliminary (**Fig D and Table F in S1 Text**).

### Effect of seawater on cercarial survival

**Fig 4:**
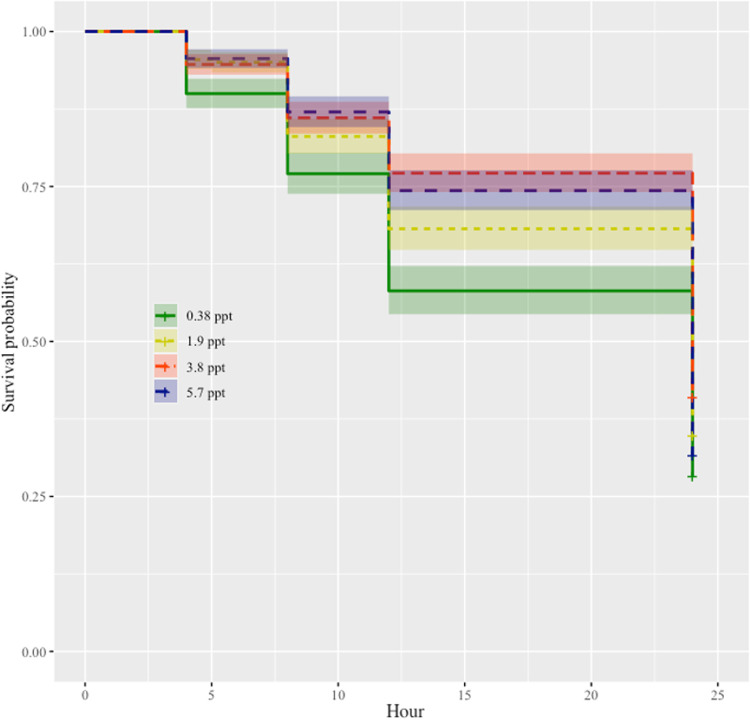
Survival probability of cercariae in 0.38 ppt, 1.9 ppt, 3.8 ppt, and 5.7 ppt salinity treatments over a 24-hour period. All four treatment groups are significantly different from one another except for 1.9 ppt and 5.7 ppt (p > 0.05) For pairwise comparisons see supplement **Table G in S1 Text**.

Cercariae in the 3.8 ppt salinity treatment had the highest survival rate. The 5.7 ppt salinity treatment had the next highest survival rate, followed by the 1.9 ppt treatment (**Fig 4**). Interestingly, the cercarial survival was lowest in the 0.38 ppt (control) treatment with this treatment having significantly lower survival than the 1.9 ppt (p < 0.05), 3.8 ppt (p < 0.0001), and 5.7 ppt treatments (p = 0.0001; For coefficients and pairwise comparisons see supplement **Table G in S1 Text**). The 1.9 ppt salinity treatment was significantly lower than the 3.8 ppt treatment (p < 0.05) but was not different from the 5.7 ppt treatment (p > 0.05). The 3.8 ppt salinity treatment was significantly higher than the 5.7 ppt treatment (p < 0.05).

## Discussion

Seawater intrusion caused by climate change is an on-going issue in the Nile Delta of Egypt. How seawater intrusion will impact freshwater parasite-host interactions and disease prevalence is of importance, especially in the transmission of human schistosomiasis. Seawater intrusion of coastal habitats and schistosome transmission co-occur across the globe (e.g., the Yangtze River Delta in China and Mekong River Delta in Vietnam)[48–50] and similar conditions likely exist for other host-parasite systems. In this study, we explored the effect of seawater on *Schistosoma mansoni* infection success in the freshwater snail *Biomphalaria alexandrina*. Experimental conditions were designed to mimic seawater intrusion as it occurs in the Nile Delta of Egypt. We investigated crucial factors that contribute to host and parasite interactions such as snail survival, snail egg mass production, infection prevalence, as well as parasite (cercarial) survival. Our results demonstrate that snail survival (**Fig 1),** snail reproduction (**Fig 2**), and snail infection prevalence (**Fig 3**) decreased as seawater concentrations increased across treatment groups. Additionally, cercarial survival showed a nonlinear response to seawater concentrations with the 0.38 ppt treatment (control) having the lowest survival while cercariae in 3.8 ppt had higher survival than those in 0.38, 1.9, and 5.7 ppt treatments (**Fig 4**).

To our knowledge, this study is the first to investigate the impact of seawater on the *Biomphalaria alexandrina*–*Schistosoma mansoni* host-parasite interaction using environmentally realistic seawater levels [51]. Previous studies have shown that the fecundity and survival of snails are adversely affected by salinities as low as 1 ppt, with significant reductions occurring between 3.5 and 4.5 ppt resulting in progressive elimination of snails [18,52]. Additionally, our result demonstrating higher cercarial survival rates at intermediate seawater concentrations is supported by previous studies, however, the salinity at which survival peaks varies among different host and parasite species and strains [36,53,54]. Despite the observed decreases in snail fitness and parasite transmission at higher seawater concentrations, production of infective cercariae proceeded successfully in concentrations of seawater up to 5.7 ppt suggesting that although parasite burden may be lessened, infections could still occur as sea levels rise [15 and the current study]. Of course, cercarial survival is not necessarily predictive of cercarial infectivity. Unfortunately, logistical constraints prevented us from assessing infectivity in vertebrate hosts. This limits our ability to fully quantify the impact rising sea levels may have on parasite transmission to humans.

We speculate that these results are caused primarily by osmotic stress and energy allocation to osmoregulation. Osmoregulation is a critical, energy-costly function of a normal cell to maintain fitness [55]. Organisms under osmotic pressure will have less energy to allocate to growth and reproduction. In our experiment, this likely led to the decrease in snail survival and snail egg mass production. Osmotic pressure may also alter snail mobility, foraging, and other ecological relationships that could influence the dispersal of infective parasite larvae. Parasite larvae, such as miracidia that infect snails, are also affected by osmotic stress in the process of finding and infecting hosts, causing lower infection prevalence as salinity increased. Some authors have suggested that in contrast to miracidia, cercariae, which infect humans, require less energy for osmoregulation due to the lower difference between external and internal salinity to a certain threshold [56]. Therefore, cercariae seem to possess a higher tolerance than their snail hosts to increasing salinity, which may drive the observed non-linear relationship between cercarial survival and seawater concentration [34 and references therein].

The current experiment was designed to examine the specific impacts of salinity on this host-parasite interaction. As such, some simplifying assumptions and limitations of this experiment exist. When salinity is altered, other factors are likely to change. Controlling for factors such as conductivity, pH, dissolved oxygen etc. independent of salinity would require multiple follow-up studies as these factors covary with salinity. In our cercarial survival experiment, the use of only cercariae from the 0.38 ppt treatment, due to low cercarial output and infection prevalence in other treatments, does not account for possible acclimatization of cercariae to treatment conditions which may influence our conclusions. In an ecological context, impacts will be contingent upon proximity to the sea and/or seawater movement into groundwater reserves. It is possible that some parasite populations in the Nile Delta have already begun to adapt to variation in salinity. There are multiple impacts of climate change on freshwater systems. Here we examined salinity increases and parasite transmission without changing other factors. We focused solely on the interactions between host and parasite, but these interactions are nested within complex food webs. Parasites and their hosts can also function as prey in an ecosystem, and the changing salinity can affect these relationships and parasite transmission [57,58]. Certainly, additional factors should be evaluated to accurately assess the future trend of schistosomiasis transmission in areas with rising sea levels. Besides salinity, climate change will also influence temperature, pH, rainfall, flooding, and drought [59]. Snail fitness is likely impacted by temperature alterations and drought, and these alterations certainly impact snail mortality and parasite production [31,60–65].

## Conclusions

Taken together, reduced survival, reproduction, and infection prevalence in snail hosts with increasing salinity will lead to lower snail population sizes and potentially fewer human schistosomiasis infections in areas with seawater intrusion. However, the ability of organisms to rapidly adapt to changing conditions cannot be overlooked. Although a decrease in human schistosomiasis might be expected, higher salt concentrations did not completely halt snail reproduction, snail infection, or cercarial release, suggesting parasites could still potentially infect humans, continue the life cycle, and adapt to alterations in salinity.

We have demonstrated that increasing concentrations of seawater in freshwater systems (such as those which may occur with rising sea levels) can have a significant impact on host-parasite interactions. Our study reveals how a single abiotic factor, salinity, can play a significant role in disease transmission. Further investigation of the role of multiple environmental factors, food web interactions, and rapid evolutionary responses of hosts and parasites to sea level rise will be needed to more accurately evaluate the future of disease transmission in these altered ecosystems.

## Supporting information

S1 Text **Fig A.** Salt Assessment was done by NMDS. Visually, the closer the labels are to the “Actual seawater”, the more similar the brand composition is to the real-life seawater. **Table A.** Salt assessment of nine brands of commercial salt through analyzing major cations, major anions, and nutrients contained. **Fig B.** Survival probability of infected snails and uninfected snails in 0.38 ppt salinity treatment group. Time is presented in weeks. The survival probability does not significantly differ between infected snails and uninfected snails in the 0.38 ppt salinity treatment group (p > 0.05) over this time period. **Table B.** Estimate, SE, t ratio, and p value of pairwise comparisons of snail survival between salinity treatments. **Table C.** Estimate, SE, t ratio, and p value of pairwise comparisons of snail egg mass production between salinity treatments. **Fig C.** Egg mass output for infected snails in treatment groups 0.38 ppt, 1.9 ppt, 3.8 ppt, and 5.7 ppt. No salinity treatments had significantly different reproductive output. The horizontal lines on the violin plot represent data quantiles of 25%, 50%, and 75%. For pairwise comparisons see supplement Table S4. **Table D** Estimate, SE, t ratio, and p value of pairwise comparisons of infected snail egg mass production between salinity treatments. **Table E.** Estimate, SE, t ratio, and p value of pairwise comparisons of snail infection prevalence between salinity treatments. **Fig D.** Cercariae output in treatment groups 0.38 ppt, 1.9 ppt, 3.8 ppt, and 5.7 ppt. The horizontal lines on the violin plot represent data quantiles of 25%, 50%, and 75%. **Table F.** Estimate, SE, t ratio, and p value of pairwise comparisons of cercariae count between salinity treatments. **Table G.** Estimate, SE, t ratio, and p value of pairwise comparisons of cercarial survival between salinity treatments.
(DOCX)
