# Correction: Opportunity or catastrophe? effect of sea salt on host-parasite survival and reproduction

**DOI:** 10.1371/journal.pntd.0010466

**Published:** 2022-05-18

**Authors:** Ao Yu, J. Trevor Vannatta, Stephanie O. Gutierrez, Dennis J. Minchella

Fig 1’s coloring is incorrect. Please see the correct Fig 1 below.

**Fig 1:**
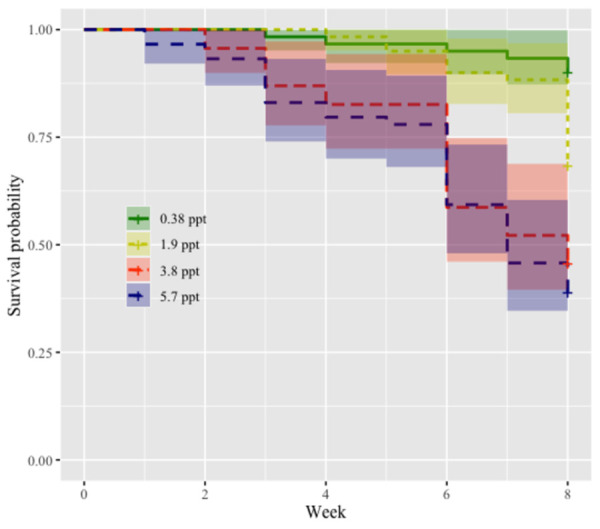
Probability of survival for snails in treatment groups of 0.38 ppt, 1.9 ppt, 3.8 ppt, 5.7 ppt salinity treatments. Snail survival in 0.38 ppt is significantly higher than 1.9 ppt (p < 0.05), 3.8 ppt (p < 0.0001), and 5.7 ppt (p < 0.0001). The survival probability of 1.9 ppt is significantly higher than 3.8 ppt and 5.7 ppt salinity treatments (p < 0.05). For coefficients and pairwise comparisons see supplement Table B in S1 Text.

## References

[1] Yu A, Vannatta JT, Gutierrez SO, Minchella DJ (2022) Opportunity or catastrophe? effect of sea salt on host-parasite survival and reproduction. PLoS Negl Trop Dis 16(2): e0009524. 10.1371/journal.pntd.0009524 35202408 PMC8870500

