# Supplementary material for: Opportunity or catastrophe? effect of sea salt on host-parasite survival and reproduction
Source: PLoS Negl Trop Dis. 2022 Feb 24;16(2):e0009524. doi: 10.1371/journal.pntd.0009524 (PMC8870500; doi:10.1371/journal.pntd.0009524)
Supplement: S1 Text — Fig A. Salt Assessment was done by NMDS. Visually, the closer the labels are to the “Actual seawater”, the more similar the brand composition is to the real-life seawater. Table A. Salt assessment of nine brands of commercial salt through analyzing major cations, major anions, and nutrients contained. Fig B. Survival probability of infected snails and uninfected snails in 0.38 ppt salinity treatment group. Time is presented in weeks. The survival probability does not significantly differ between infected snails and uninfected snails in the 0.38 ppt salinity treatment group (p > 0.05) over this time period. Table B. Estimate, SE, t ratio, and p value of pairwise comparisons of snail survival between salinity treatments. Table C. Estimate, SE, t ratio, and p value of pairwise comparisons of snail egg mass production between salinity treatments. Fig C. Egg mass output for infected snails in treatment groups 0.38 ppt, 1.9 ppt, 3.8 ppt, and 5.7 ppt. No salinity treatments had significantly different reproductive output. The horizontal lines on the violin plot represent data quantiles of 25%, 50%, and 75%. For pairwise comparisons see supplement Table S4. Table D Estimate, SE, t ratio, and p value of pairwise comparisons of infected snail egg mass production between salinity treatments. Table E. Estimate, SE, t ratio, and p value of pairwise comparisons of snail infection prevalence between salinity treatments. Fig D. Cercariae output in treatment groups 0.38 ppt, 1.9 ppt, 3.8 ppt, and 5.7 ppt. The horizontal lines on the violin plot represent data quantiles of 25%, 50%, and 75%. Table F. Estimate, SE, t ratio, and p value of pairwise comparisons of cercariae count between salinity treatments. Table G. Estimate, SE, t ratio, and p value of pairwise comparisons of cercarial survival between salinity treatments. (DOCX) [file pntd.0009524.s001.docx]

**S1 Text**

We assessed various sea salt brands for use in this experiment[1] (**Table A**). The data was analyzed by non-metric multidimensional scaling (NMDS) in the vegan package in R to compare with true seawater mineral concentrations (**Fig A**). We chose the brand most similar to true seawater that was available to us (Instant Ocean Sea Salt).


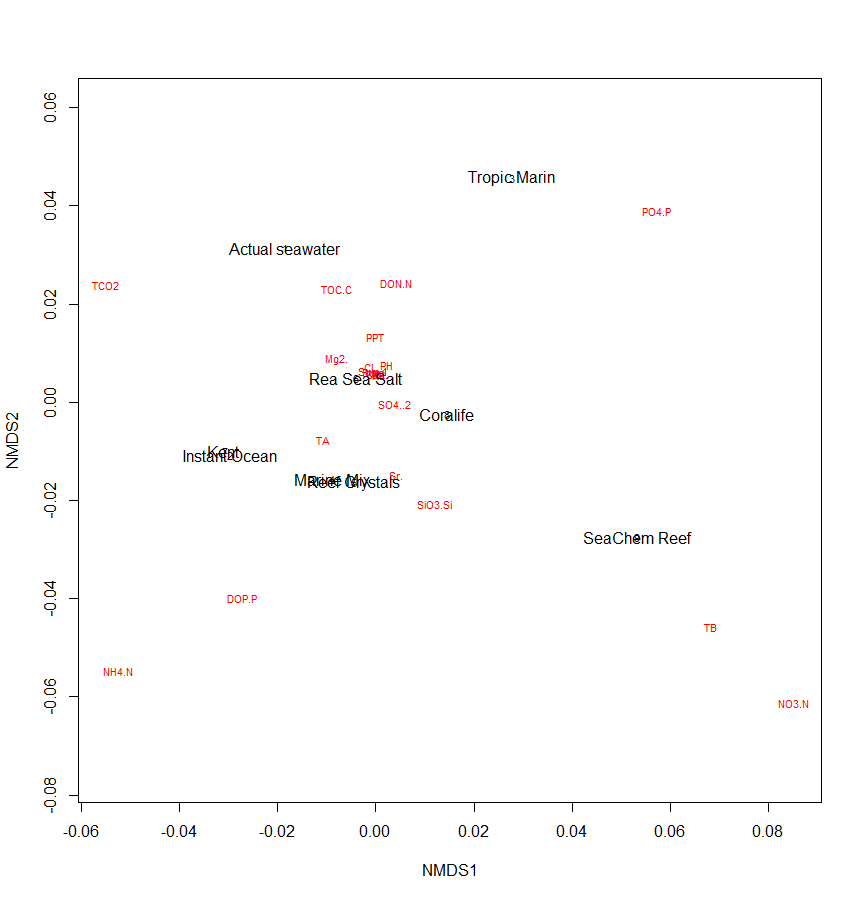


**Fig A.** Salt Assessment was done by NMDS. Visually, the closer the labels are to the “Actual seawater”, the more similar the brand composition is to the real-life seawater.

| **Substance** | **Actual seawater** | **Instant Ocean** | **Tropic Marin** | **Marine Mix** | **Reef Crystals** | **Rea Sea Salt** | **Kent** | **Coralife** | **SeaChem Reef** |
| --- | --- | --- | --- | --- | --- | --- | --- | --- | --- |
| PPT | 35 | 29.65 | 32.64 | 29.4 | 28.91 | 30.07 | 28.85 | 28.39 | 29.54 |
| **Major Cations (mmol**  **kg-1)** |  |  |  |  |  |  |  |  |  |
| Na+ | 470 | 462 | 442 | 467 | 461 | 472 | 460 | 464 | 504 |
| K+ | 10.2 | 9.4 | 9.1 | 10.1 | 9.5 | 9.9 | 10.1 | 9.3 | 10.7 |
| Mg2+ | 53 | 52 | 46 | 53 | 50 | 55 | 57 | 63 | 37 |
| Ca2+ | 10.3 | 9.4 | 9.1 | 10.1 | 9.5 | 9.9 | 10.1 | 9.3 | 10.7 |
| Sr+ | 0.09 | 0.19 | 0.08 | 0.15 | 0.08 | 0.1 | 0.1 | 0.08 | 0.21 |
| Sum | 607 | 594 | 561 | 601 | 589 | 610 | 605 | 620 | 609 |
| **Major Anions (mmol**  **kg-1)** |  |  |  |  |  |  |  |  |  |
| Cl- | 550 | 521 | 497 | 538 | 520 | 537 | 531 | 566 | 516 |
| SO4 -2 | 28 | 23 | 21 | 28 | 27 | 25 | 24 | 15 | 37 |
| TCO2 | 1.9 | 1.9 | 1.1 | 2.1 | 0.75 | 1.08 | 2.52 | 0.32 | 0.12 |
| TB | 0.42 | 0.44 | 0.36 | 0.41 | 0.65 | 0.54 | 0.54 | 1.26 | 4.9 |
| Sum | 608 | 569 | 541 | 596 | 574 | 588 | 582 | 597 | 595 |
| **Nutrients (µmol**  **kg-1)** |  |  |  |  |  |  |  |  |  |
| PO4:P | 0.2 | 0.05 | 1.2 | 0.46 | 0.32 | 0.37 | 0.16 | 0.95 | 0.57 |
| NO3:N | 0.2 | 1 | 2.2 | 1.63 | 5 | 0.79 | 2.05 | 2.3 | 18.4 |
| NH4:N | 0.2 | 10.2 | 0.55 | 9.2 | 7.8 | 5.2 | 11.9 | 8.4 | 0.7 |
| SiO3:Si | 5 | 4.2 | 3.2 | 11.5 | 5.9 | 4.5 | 4.1 | 2.7 | 11.3 |
| DOP:P | 0.2 | 0.1 | 0 | 0.2 | 0.2 | 0.1 | 0.1 | 0.2 | 0.1 |
| DON:N | 10 | 2.9 | 5.5 | 8.2 | 6.3 | 1.9 | 2.4 | 11.2 | 3.1 |
| TOC:C | 50 | 29 | 32 | 29 | 28 | 29 | 28 | 28 | 22 |
| PH | 8.25 | 8.35 | 8.9 | 8.49 | 9.28 | 8.69 | 8.08 | 9.17 | 8.81 |
| TA | 2.3 | 2.3 | 1.5 | 3.1 | 3.2 | 1.6 | 2.7 | 1.5 | 2.2 |

**Table A.**  Salt assessment of nine brands of commercial salt through analyzing major cations, major anions, and nutrients contained[1].


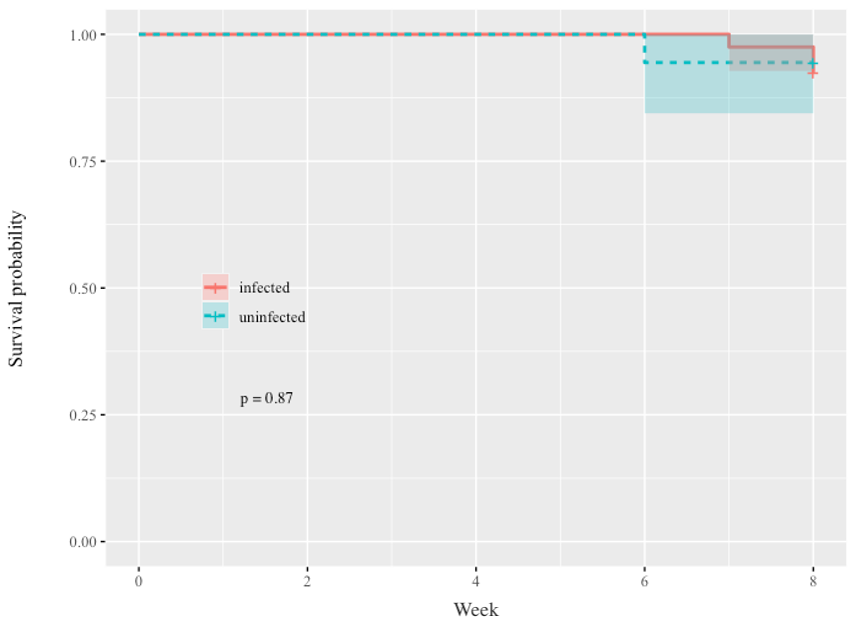


**Fig B.** Survival probability of infected snails and uninfected snails in 0.38 ppt salinity treatment group. Time is presented in weeks. The survival probability does not significantly differ between infected snails and uninfected snails in the 0.38 ppt salinity treatment group (p > 0.05) over this time period.

| **Contrast** | **Estimate** | **SE** | **t Ratio** | **p Value** |
| --- | --- | --- | --- | --- |
| **0.38 ppt – 1.9 ppt** | 0.152 | 0.107 | 1.414 | 0.4797 |
| **0.38 ppt – 3.8 ppt** | 0.373 | 0.126 | 2.954 | 0.0162 |
| **0.38 ppt – 5.7 ppt** | 0.979 | 0.175 | 5.589 | <.0001 |
| **3.8 ppt – 1.9 ppt** | -0.222 | 0.107 | -2.070 | 0.1569 |
| **5.7 ppt – 1.9 ppt** | -0.827 | 0.161 | -5.126 | <.0001 |
| **3.8 ppt – 5.7 ppt** | 0.605 | 0.174 | 3.486 | 0.0027 |

**Table B.** Estimate, SE, t ratio, and p value of pairwise comparisons of snail survival between salinity treatments.

| **Contrast** | **Estimate** | **SE** | **t Ratio** | **p Value** |
| --- | --- | --- | --- | --- |
| **0.38 ppt – 1.9 ppt** | -1.216 | 0.468 | -2.594 | 0.0435 |
| **0.38 ppt – 3.8 ppt** | -2.059 | 0.455 | -4.524 | <.0001 |
| **0.38 ppt – 5.7 ppt** | -2.235 | 0.422 | -5.058 | <.0001 |
| **3.8 ppt – 1.9 ppt** | 0.844 | 0.305 | 2.767 | 0.0269 |
| **5.7 ppt – 1.9 ppt** | 1.019 | 0.284 | 3.548 | 0.0017 |
| **3.8 ppt – 5.7 ppt** | -0.176 | 0.260 | -0.674 | 0.9032 |

**Table C.** Estimate, SE, t ratio, and p value of pairwise comparisons of snail egg mass production between salinity treatments.


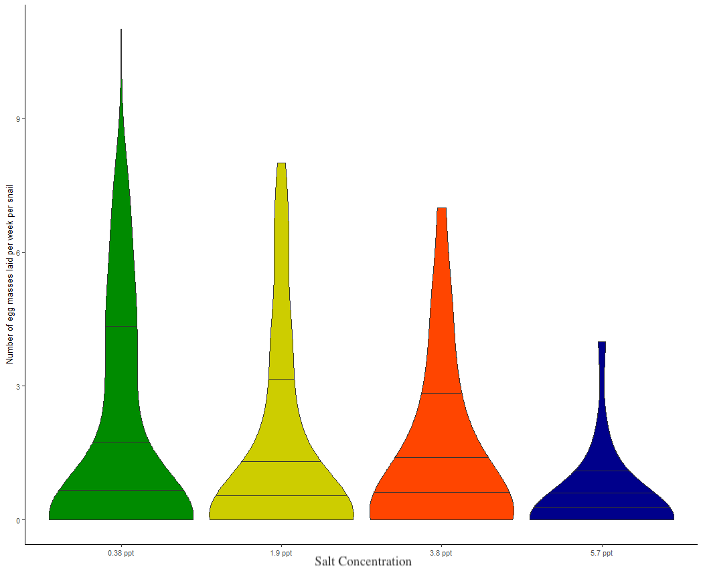


**Fig C.** Egg mass output for infected snails in treatment groups 0.38 ppt, 1.9 ppt, 3.8 ppt, and 5.7 ppt. No salinity treatments had significantly different reproductive output. The horizontal lines on the violin plot represent data quantiles of 25%, 50%, and 75%. For pairwise comparisons see supplement Table S4.

| **Contrast** | **Estimate** | **SE** | **t Ratio** | **p Value** |
| --- | --- | --- | --- | --- |
| **0.38 ppt – 1.9 ppt** | 0.266 | 0.182 | 1.243 | 0.5633 |
| **0.38 ppt – 3.8 ppt** | 0.437 | 0.268 | 1.630 | 0.3256 |
| **0.38 ppt – 5.7 ppt** | 1.356 | 0.807 | 1.681 | 0.2988 |
| **3.8 ppt – 1.9 ppt** | -0.211 | 0.310 | -0.681 | 0.8908 |
| **5.7 ppt – 1.9 ppt** | -1.130 | 0.819 | -1.380 | 0.4738 |
| **3.8 ppt – 5.7 ppt** | 0.919 | 0.840 | 1.094 | 0.6610 |

**Table D.** Estimate, SE, t ratio, and p value of pairwise comparisons of infected snail egg mass production between salinity treatments.

| **Contrast** | **Estimate** | **SE** | **t Ratio** | **p Value** |
| --- | --- | --- | --- | --- |
| **0.38 ppt – 1.9 ppt** | 2.451 | 0.461 | 5.312 | <0.0001 |
| **0.38 ppt – 3.8 ppt** | 2.686 | 0.558 | 4.817 | <0.0001 |
| **0.38 ppt – 5.7 ppt** | 3.076 | 0.597 | 5.154 | <0.0001 |
| **3.8 ppt – 1.9 ppt** | -0.234 | 0.602 | -0.389 | 0.9795 |
| **5.7 ppt – 1.9 ppt** | -0.624 | 0.639 | -0.978 | 0.7578 |
| **3.8 ppt – 5.7 ppt** | 0.390 | 0.771 | 0.549 | 0.9457 |

**Table E.** Estimate, SE, t ratio, and p value of pairwise comparisons of snail infection prevalence between salinity treatments.


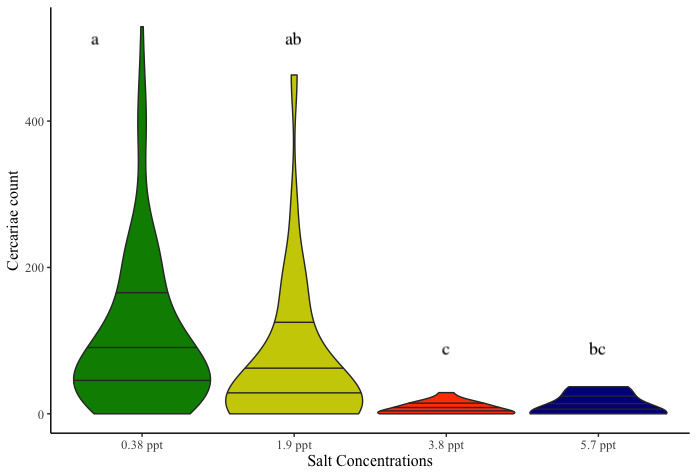


**Fig D.** Cercariae output in treatment groups 0.38 ppt, 1.9 ppt, 3.8 ppt, and 5.7 ppt. The horizontal lines on the violin plot represent data quantiles of 25%, 50%, and 75%.

| **Contrast** | **Estimate** | **SE** | **t Ratio** | **p Value** |
| --- | --- | --- | --- | --- |
| **0.38 ppt – 1.9 ppt** | 0.247 | 0.252 | 0.978 | 0.7458 |
| **0.38 ppt – 3.8 ppt** | 2.172 | 0.367 | 5.912 | <0.0001 |
| **0.38 ppt – 5.7 ppt** | 1.370 | 0.451 | 3.035 | 0.0130 |
| **3.8 ppt – 1.9 ppt** | -1.925 | 0.429 | -4.491 | <0.0001 |
| **5.7 ppt – 1.9 ppt** | -1.123 | 0.504 | -2.229 | 0.1086 |
| **3.8 ppt – 5.7 ppt** | -0.802 | 0.563 | -1.425 | 0.4629 |

**Table F.** Estimate, SE, t ratio, and p value of pairwise comparisons of cercariae count between salinity treatments.

| **Contrast** | **Estimate** | **SE** | **t Ratio** | **p Value** |
| --- | --- | --- | --- | --- |
| **0.38 ppt – 1.9 ppt** | 0.277697 | 0.0676 | 4.108 | 0.0004 |
| **0.38 ppt – 3.8 ppt** | 0.540601 | 0.0693 | 7.806 | <0.0001 |
| **0.38 ppt – 5.7 ppt** | 0.278454 | 0.0664 | 4.193 | 0.0001 |
| **3.8 ppt – 1.9 ppt** | 0.262904 | 0.0688 | 3.819 | 0.0007 |
| **5.7 ppt – 1.9 ppt** | 0.000757 | 0.0660 | 0.011 | 1.0000 |
| **3.8 ppt – 5.7 ppt** | -0.262147 | 0.0675 | 3.882 | 0.0007 |

**Table G.** Estimate, SE, t ratio, and p value of pairwise comparisons of cercarial survival between salinity treatments.

**Reference**

1 Atkinson, M. J. and C. Bingman. 1997. Elemental composition of commercial

seasalts. Journal of Aquariculture and Aquatic Sciences 8, 2: 39 – 43
